# The genus *Saissetia* (Hemiptera, Coccomorpha, Coccidae) in China, with description of a new species

**DOI:** 10.3897/zookeys.873.36661

**Published:** 2019-08-29

**Authors:** Tong Cao, Na Zhang, Jinian Feng

**Affiliations:** 1 Key Laboratory of Plant Protection Resources and Pest Management, Ministry of Education, Entomological Museum, College of Plant Protection, Northwest A&F University, Yangling, Shaanxi Province, 712100, China Northwest A&F University Yangling China

**Keywords:** Coccoidea, plant pest, soft scales, taxonomy

## Abstract

*Saissetia kunmingensis* Cao & Feng, **sp. nov.**, a member of the genus *Saissetia* Kanda, 1960, is a new species in China that is described and illustrated in this study, based on adult females. This species was found on *Osmanthus fragrans* (Oleaceae) plants from Kunming, Yunnan Province (China). An updated key to females of the eight species of *Saissetia* which occur in China was developed.

## Introduction

Soft scale insects (Hemiptera, Coccomorpha, Coccidae) are the third largest family in the Coccomorpha, followed by the Diaspididae (armored scales) and the Pseudococcidae (mealybugs) (García Morales et al. 2016). Many species of soft scales are distributed in many countries throughout the world and considered to be important pests on agricultural and horticultural crops as well as ornamental plants (Henderson and Hodgson 2005). In China, they are also important pests on all of these types of plants (Yang 1982).

Prior to this study, the genus *Saissetia* Deplanche consisted of 45 known species in the world of which seven species had been reported from China (García Morales et al. 2016). In this study, we describe and illustrate a new species *Saissetia kunmingensis* sp. nov. from China. This new species shares certain characteristics with *S. coffeae* (Walker) and *S. oleae* (Olivier). An updated key to adult females of the eight species in the genus *Saissetia* known to occur in China is provided.

## Materials and methods

**Figure 1.**
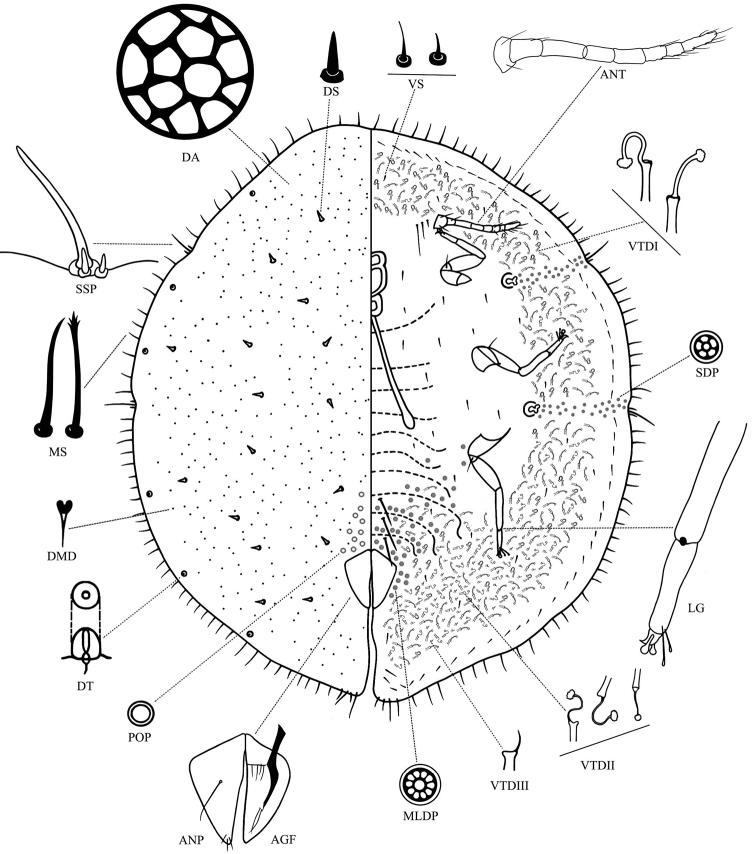
*Saissetia kunmingensis* Cao & Feng, sp. nov., adult female. The dorsal surface is depicted on the left side and the ventral surface on the right side, with some important characters shown enlarged around the main illustration. Abbreviations: **AGF** ano-genital fold **ANP** anal plates **ANT** antenna **DMD** dorsal microduct **DS** dorsal seta **DT** dorsal tubercles **LG** tibio-tarsus of hind leg **MS** marginal setae **MLDP** multilocular disc-pore **POP** preopercular pores **SDP** spiracle disc-pore **SSP** stigmatic spines **VTD** ventral tubular ducts of type I–III **VS** ventral setae.

**Figure 2.**
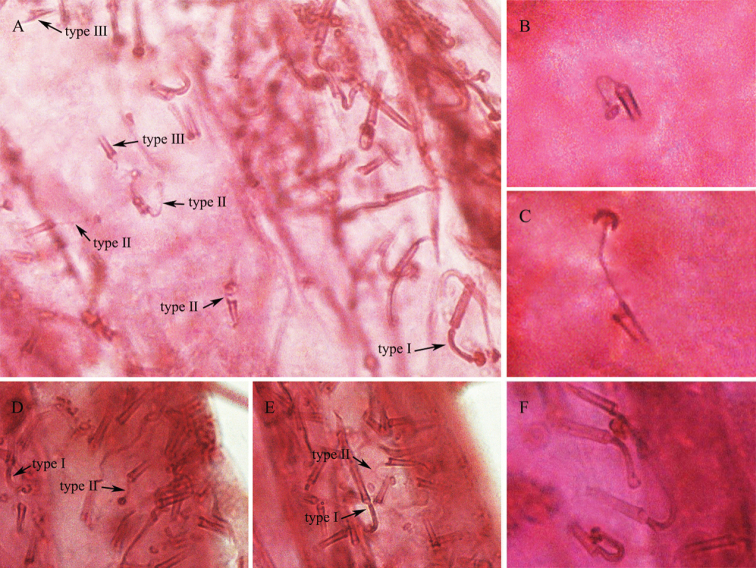
Ventral tubular ducts of *Saissetia kunmingensis* Cao & Feng, sp. nov. **A** type I, type II and type III VTDs present submarginally on posterior abdominal segments **B** type II VTD present submarginally on posterior abdominal segments **C** type II VTD present mediolaterally on posterior abdominal segments **D** type I and type II VTDs present submarginally around body **E** type I and type II VTDs present submarginally around body **F** type I VTDs present mediolaterally on posterior abdominal segments.

Scale insect samples were collected from leaves and twigs of *Osmanthus fragrans* (Thunb.) Lour. (Oleaceae) in Yunnan Province in China and then stored in envelopes. Specimens were immersed in chloroform to remove wax secretions before the preparation of slides. Slides were mounted using methods described by Henderson and Hodgson (2000). Insect morphology was observed under an EVOS digital inverted microscope. The illustration of the adult female (Fig. 1) was drawn with an Olympus BH-2 stereoscopic microscope. In the illustration, the dorsum is depicted on the left side and the venter on the right side and important characters are shown around the main illustration. Photographs of ventral tubular ducts of young adult females (Fig. 2) were taken by an EVOS digital inverted microscope and a Nikon ECLIPSE Ti-U microscope. Photographs were enhanced with Adobe Photoshop CS6. All measurements were made using NIT-Elements D software and are presented in micrometers (μm) or millimeters (mm).

All specimens were deposited in the Northwest A&F University, Yangling, Shaanxi, China (**NWAFU**).

## Taxonomy

### Genus Saissetia

Taxon classificationAnimaliaHemipteraCoccidae

Deplanche, 1859

436869818ABB5E3883AC8CEEF9F0BC9B

#### Type-species.

*Saissetia coffeae* (Walker, 1852).

*Saissetia* Deplanche, 1859: 6; Newstead 1917: 361; Hall 1935: 73; De Lotto 1956: 239; De Lotto 1957: 170; De Lotto 1963: 191; De Lotto 1965: 219; Tang 1991: 215; Henderson and Hodgson 2000: 208; Choi and Lee 2017: 101; Zhang et al. 2018: 97.

#### Generic diagnosis.

**Adult female.** Body dorsum convex, H-shaped ridge present medially on dorsum. Derm with sclerotized reticulations and dermal areolations becoming well developed in older females; dorsal setae conical, thick and sturdy; submarginal tubercles present or absent on submarginal area; dorsal tubular ducts absent; anal plates triangular, together quadrate, each with a distinct discal seta; some discal setae with a sharp and pointed apex, or with a fimbriate and frayed apex. Marginal setae slender or curved, spinose with pointed apexes, bifid, frayed, or fimbriate and branched, all with well-developed basal sockets. Legs well developed, each with or without a well-developed tibio-tarsal articulatory sclerosis; claw without a denticle; multilocular disc-pores with 10–12 loculi, usually 10, present in vulvar area and on anterior abdominal segments, some sparsely present on thorax; ventral tubular ducts (VTDs) present in a broad submarginal band; 1 to 4 types of VTDs, including type I: ducts with a long outer ductule and a narrow inner ductule, width of inner ductule half or less than half width of outer ductule, with a flower-shaped terminal gland; type II: ducts with a moderately long outer ductule and a long, extremely narrow inner ductule with a well-developed terminal gland; type III: a short, small duct with a filament-like inner ductule without a glandular end; type IV: a duct with a broad inner ductule of similar width and length as the outer ductule, with a well-developed terminal gland.

### Saissetia kunmingensis

Taxon classificationAnimaliaHemipteraCoccidae

Cao & Feng sp. nov.

9902C0FA0C2C567D8B7B84E53DEED710

http://zoobank.org/8EDE0261-9C80-4CA2-86CF-E8B24EB1FDB8

#### Material examined.

**Holotype**: adult female. Kunming, Yunnan Province, China. 25°04'N, 102°42'E. 1.vii.2018, on *Osmanthus fragrans* (Oleaceae), Cao Tong, Zhang Na (NWAFU). **Paratypes**: one adult female on the same slide with holotype, in the lower left corner, 4 adult females on another 2 slides, each slide with 2 specimens, data same as holotype.

#### Diagnosis.

The adult females of *S. kunmingensis* sp. nov. can be diagnosed by a combination of the following features: (1) body almost circular to broad oval; (2) derm with sclerotized reticulations well developed; (3) dorsal submarginal tubercles convex, one or two between anterior stigmatic clefts, one or two between anterior and posterior stigmatic clefts, and three or four between each posterior stigmatic cleft and anal cleft; (4) dorsal tubular ducts absent; (5) dorsal setae conical, thick and spinose; (6) anal plates each with a distinct long discal setae with a sharp and pointed apex; (7) marginal setae slender, straight, mostly with a toothed apex, occasionally with a simple pointed apex; (8) antennae with 8 segments; (9) spiracles normal, with a distinct sclerotic plate, rarely indistinct; (10) legs with tibio-tarsal articulation and a small tibio-tarsal articulatory sclerosis; (11) multilocular disc-pores usually have 10 loculi, occasionally 11, present around vulva, across mediolateral of all abdominal segments, a few present laterad to metacoxa, sometimes present on thorax; (12) three types of VTDs (for descriptions and distributions of these see species description).

#### Description.

**Appearance of live insects.** Insects yellow brown, mature adult females dark brown and reddish brown. Body broadly oval, almost circular. Dorsum of mature adults strongly sclerotized, distinctly convex with H-shaped ridge present.

**Slide-mounted adult female.** (Fig. 1) Body broadly oval, almost circular, broadest in anterior abdomen and thorax; body length 2.0–3.0 mm, width 1.5–2.0 mm. Anal cleft approximately 1/7–1/6 of the body length.

**Dorsum.** Derm with cell-like and polygonous clear areas (areolations), sclerotized reticulations on mature insects. Dorsal setae conical and sturdy, each with a pointed apex and a well-developed basal socket, scattered on dorsum, total length about 20.1–28.9 μm long (or 14.2–21.0 μm long excluding a basal socket). Submarginal tubercles numbering 1 or 2 between anterior stigmatic clefts on head, 1 or 2 between anterior and posterior stigmatic clefts, and 3 or 4 between each posterior stigmatic cleft and anal cleft. Dorsal microducts distinctly present in cell-like clear areas. Dorsal tubular ducts absent. Preopercular pores distinct, slightly convex and circular, present in front of anal plates, numbering 16–20. Anal plates each triangular, together quadrate, 240.8–267.2 μm long, 211.9–229.6 μm wide, anterolateral margin slightly convex, 118.9–140.3 μm long, posterolateral margin slightly convex, 208.3–215.6 μm long, posterior margin longer than anterior margin, outer angle slightly obtuse; plates with a well-developed supporting bar, a distinct long discal seta with a sharp and pointed apex, and 3 apical setae. Ano-genital fold with 4 pairs of anterior margin setae, 42.1–47.2 μm long and 2 lateral marginal setae, 70.9–74.6 μm long. Anal ring subcircular, with 8 anal ring setae.

**Margin.** Marginal setae 66.9–80.6 μm long, with well-developed basal sockets, mostly slender and straight, with a frayed, fimbriate and branched apex, but a few marginal setae with sharp and rather bluntly pointed apices; with 38–44 setae between anterior stigmatic clefts, 9–10 setae between anterior and posterior stigmatic clefts on each side, and 27–31 setae between the posterior stigmatic cleft and anal cleft. Stigmatic clefts not deep but distinct, each cleft containing three slender, tapered and bluntly spinose stigmatic spines, with well-developed basal sockets; median spine longest, 129.8–133.9 μm long, about 5 to 6 times as long as the lateral spines, each 26.2–29.0 μm long.

**Venter.** Derm membranous. Antennae with 8 segments, rarely 7 segments, total antennal length 453.8–463.4 μm; segment III longest. Usually 3 pairs of setae present between antennae near their base, 1 pair of short inner setae and 2 pairs of long outer setae. Three pairs of long pregenital setae present, 160.8–183.5 μm long. Other ventral setae setose, short and fine, quite sparsely distributed, 8.4–17.1 μm long. Submarginal setae 22.7–37.5 μm long, present in a single row around body. Legs well developed, each with tibio-tarsal articulation and a tibio-tarsal articulatory sclerosis which are rarely absent, tibia 154.8–176.4 μm long, longer than tarsus, which is 119.6–128.9 μm long. Claw without a denticle; claw digitules broad and expanded at apex, about 32.0–32.4 μm long. Tarsal digitules longer than claw digitules, slender, knobbed, expanded at apex, about 48.6–54.9 μm long. Spiracles normal, with a distinct sclerotic plate (rarely indistinct). Spiracular disc-pores mostly with five loculi in the outer ring; spiracular pore bands narrow, each 3–4 rows wide. Anterior spiracular pore band with 11–19 pores, posterior spiracular pore band with 12–20 pores. Multilocular disc-pores each primarily with 10 loculi, occasionally 11, present around vulva, becoming progressively less frequent anteriorly, but present across mediolateral areas of all abdominal segments, a few present laterad to metacoxa, sometimes present on thorax. Ventral tubular ducts (VTDs) present, of three types; I, II and III (Fig. 2). Type I ducts have a rather long, broad outer ductule, 23.9–28.5 μm long, and a narrow inner ductule of similar length, 24.9–29.8 μm long, width of inner ductule being half or less than half of width of outer ductule, with a well-developed flower-shaped terminal gland; type I ducts are present submarginally in a broad band around the body, and mediolaterally on posterior abdominal segments. Type II ducts have a slightly short, broad outer ductule, 13.2–20.8 μm long, and an extremely narrow filament-like inner ductule longer than the outer ductule, 17.1–31.6 μm long, with a well-developed terminal gland; type II ducts are located submarginally and mediolaterally on posterior abdominal segments, becoming sparse, few and discrete between 2 spiracular pore bands and on the anterior of the head. Type III ducts have a short outer ductule, 7.4–11.1 μm long, and a fine inner filament-like ductule without any terminal gland; these are distributed submarginally on posterior abdominal segments, and rarely on the outer submarginal area of the head.

#### Etymology.

The species epithet *kunmingensis* refers to the place where this new species was collected, i.e., the city of Kunming.

#### Host plant.

*Osmanthus fragrans* (Thunb.) Lour.

#### Distribution.

Yunnan Province (China).

##### Key to adult females of Saissetia species occurring in China

**Table:** 

1	Each stigmatic cleft containing 3 stigmatic spines	**2**
–	Each stigmatic cleft containing 4–7 stigmatic spines	***Saissetia vivipara* Williams & Watson, 1990**
2	One type of ventral tubular duct (VTD) present	**3**
–	More than one type of VTD present	**5**
3	Legs with tibio-tarsal articulation and articulatory sclerosis	**4**
–	Legs with tibio-tarsal articulation but without articulatory sclerosis	***Saissetia neglecta* De Lotto, 1969**
4	Mature adults red; spiracle with a sclerotic plate; VTDs with enlarged inner ductules; marginal setae mostly sharp, setose and pointed	***Saissetia bobuae* Takahashi, 1935**
–	Mature adults dark brown to black; spiracle without a sclerotic plate; VTDs with narrow inner ductules; marginal setae mostly strongly frayed, fimbriate and branched	***Saissetia miranda* (Cockerell and Parrott in Cockerell 1899)**
5	VTDs of three or four types present; legs with tibio-tarsal articulation and articulatory sclerosis; discal setae sharp and pointed	**6**
–	VTDs of two types present (types I and III); legs with tibio-tarsal articulation but without articulatory sclerosis; discal setae frayed and fimbriate	***Saissetia oleae* (Olivier, 1791)**
6	VTDs present with enlarged inner ductules; VTDs with a filament-like inner ductule (type III) present in submarginal band; multilocular disc-pores present laterad to each metacoxa and mesocoxa	**7**
–	No VTDs with enlarged inner ductules; VTDs with a filament-like inner ductule (type III) present at posterior end near vulvar area; multilocular disc-pores absent from near each mesocoxa	***Saissetia kunmingensis* Cao & Feng, sp. nov.**
7	VTDs of three types present (types II, III and IV); 3 pairs of setae on ano-genital fold	***Saissetia coffeae* (Walker, 1852)**
–	VTDs of four types present (types I to IV); 4 or 5 pairs of setae on ano-genital fold	***Saissetia puerensis* Zhang & Feng in Zhang et al., 2018**

##### Morphological separation of *S. kunmingensis* sp. nov. from *S. coffeae* and *S. oleae*

*Saissetia kunmingensis* sp. nov. is morphologically similar to *S. coffeae*, which also has three types of VTDs, but these can be separated by the features shown in Table 1. (For descriptions of the types of VTDs see the generic diagnosis).

**Table 1.** Morphological features that can be used to separate *S. kunmingensis* sp. nov. and *S. coffeae*. VTDs = ventral tubular ducts.

Morphological features	*S. kunmingensis* sp. nov.	*S. coffeae*
Type I VTDs	Present submarginally in a broad band around body, mediolaterally on posterior abdominal segments	Absent
Type II VTDs	Present submarginally and mediolaterally on posterior abdominal segments, sparse, few and discrete between 2 spiracular pore bands and head	Present on inner submarginal area around body and medial thorax (Tang 1991; Choi and Lee 2017)
Type III VTDs	Present submarginally and inner submarginally on posterior abdominal segments, rarely present on submarginal area of head	Present on outer submarginal area around body (Tang 1991; Choi and Lee 2017)
Type IV VTDs	Absent	Present on medial submarginal area around body (Tang 1991; Choi and Lee 2017)
Distribution of VTDs	Distributed irregularly, type I and type II ventral tubular ducts mixed mediolaterally on posterior abdominal segments, and in a broad band around body, type III ducts mixed with them submarginally on posterior abdominal segments	Distributed regularly (Tang 1991; Choi and Lee 2017)
Dorsal setae	Thick and long, 20.1–28.9 μm long including basal socket, 14.2–21.0 μm long without a basal socket	Rather short, 6.0–9.0 μm long (Choi and Lee 2017)

*Saissetia kunmingensis* sp. nov. also has similar morphology to *S. oleae*. The morphological features that can be used to separate these two species are shown in Table 2.

**Table 2.** Morphological features that can be used to separate *S. kunmingensis* sp. nov. from *S. oleae*. VTDs = ventral tubular ducts.

Morphological features	*S. kunmingensis* sp. nov.	*S. oleae*
Type I VTDs	Present submarginally in a broad band around body, mediolaterally on posterior abdominal segments	Only present on submarginal area (Tang 1991; Henderson and Hodgson 2000)
Type II VTDs	Present submarginally and mediolaterally on posterior abdominal segments, sparse and discrete between two spiracular pore bands and on the top of anterior head	Absent
Type III VTDs	Present on submarginal posterior abdominal segments, rarely present on outer submarginal area of head	Only present mediolaterally on posterior abdominal segments (Henderson and Hodgson 2000)
Anal plates	Posterior margin much longer than anterior margin	Length of posterior margin almost equal to or slightly longer than anterior margin (Tang 1991; Hodgson 1994)
Marginal setae	38–44 setae between anterior stigmatic clefts on head; mostly fimbriate	15–30 setae between anterior stigmatic clefts on head; mostly sharp (Henderson and Hodgson 2000)

## Discussion

The new species is at present only known from Kunming, Yunnan Province. Further studies should be conducted to explore the distribution of this new species and other *Saissetia* species, especially in the north-east region of China. The only host plant found in this study was *Osmanthus fragrans*, so the host range also needs to be further studied. *Saissetia kunmingensis* sp. nov. is potentially an important pest in China. The adult females infest branches, twigs and leaves, and glassy wax and honeydew secreted by this species can make the leaves adhere to each other, which can lead to decay or even death of the host plants.

## Supplementary Material

XML Treatment for Saissetia

XML Treatment for Saissetia kunmingensis
